# Optical properties and impedance spectroscopy analyses for microscale Si pillar solar cells

**DOI:** 10.1016/j.dib.2017.07.016

**Published:** 2017-07-14

**Authors:** Malkeshkumar Patel, Joondong Kim

**Affiliations:** Photoelectric and Energy Device Applications Lab (PEDAL) and Department of Electrical Engineering, Incheon National University, 119 Academy Rd. Yeonsu, Incheon 406772, Republic of Korea

**Keywords:** Si pillars, Optical properties, Impedance spectroscopy, Reflectance profiles

## Abstract

In this data article, optical properties and impedance spectroscopy analyses were applied for the 5 μm-height pillar Si solar cells to analyzed the insight of the Si geometric effect (Yadav et al., 2017) [1]. The surface reflectance data measured for all Si pillar samples (Fixed height of 5 μm with varying width and period. Geometric features of Si pillars are summarized in Table 1) are presented. Statistical data after analysis are summarized in the table, to profile the integrated reflectance quantitatively. Impedance spectroscopy analyses of all the samples were performed to demonstrate the bias-dependent space charge region. Mott–Schottky investigation shows the enhancement of built-in potential values due to the pillar structures.

## Specifications Table

**Table:** 

Subject area	*Physics, Electrical Engineering*
More specific subject area	*Solar cells*
Type of data	*Figures, Table*
How data was acquired	*UV-visible spectrophotometer (UV-2600, Shimadzu), Potentiostat/Galvanostat (ZIVE SP1, WonA Tech, Korea)*
Data format	*Analyzed*
Experimental factors	*Optical Reflectance: 5 μm-height pillar Si solar cells* *Impedance spectroscopy: Frequency range 1 MHz to 1 Hz* *Bias range → −0.7 V to 0.4 V in step of 0.1 V* *Mott-Schottky: Frequency* → *20 kHz* *Bias range → −0.8 V to 0.4 V*
Experimental features	*Realizing high-performing Si solar cells by using periodic structures*
Data source location	*Incheon National University, Incheon-406772, Korea*
Data accessibility	*The data are with this article*

## Value of the data

• Area under the curve of reflectance of the Si microscale pillar solar cells would be useful to estimate the overall reflectance quantitatively; this analysis could be applicable to efficient anti reflectance coating researches.
• The bias dependent impedance spectra revealed the functional modulation of the space charge region of Si pillar solar cells.
• The Mott–Schottky measurement demonstrates the enhanced built-in potential according to the pillar structures.

## 1 Data

**Fig. 1:**
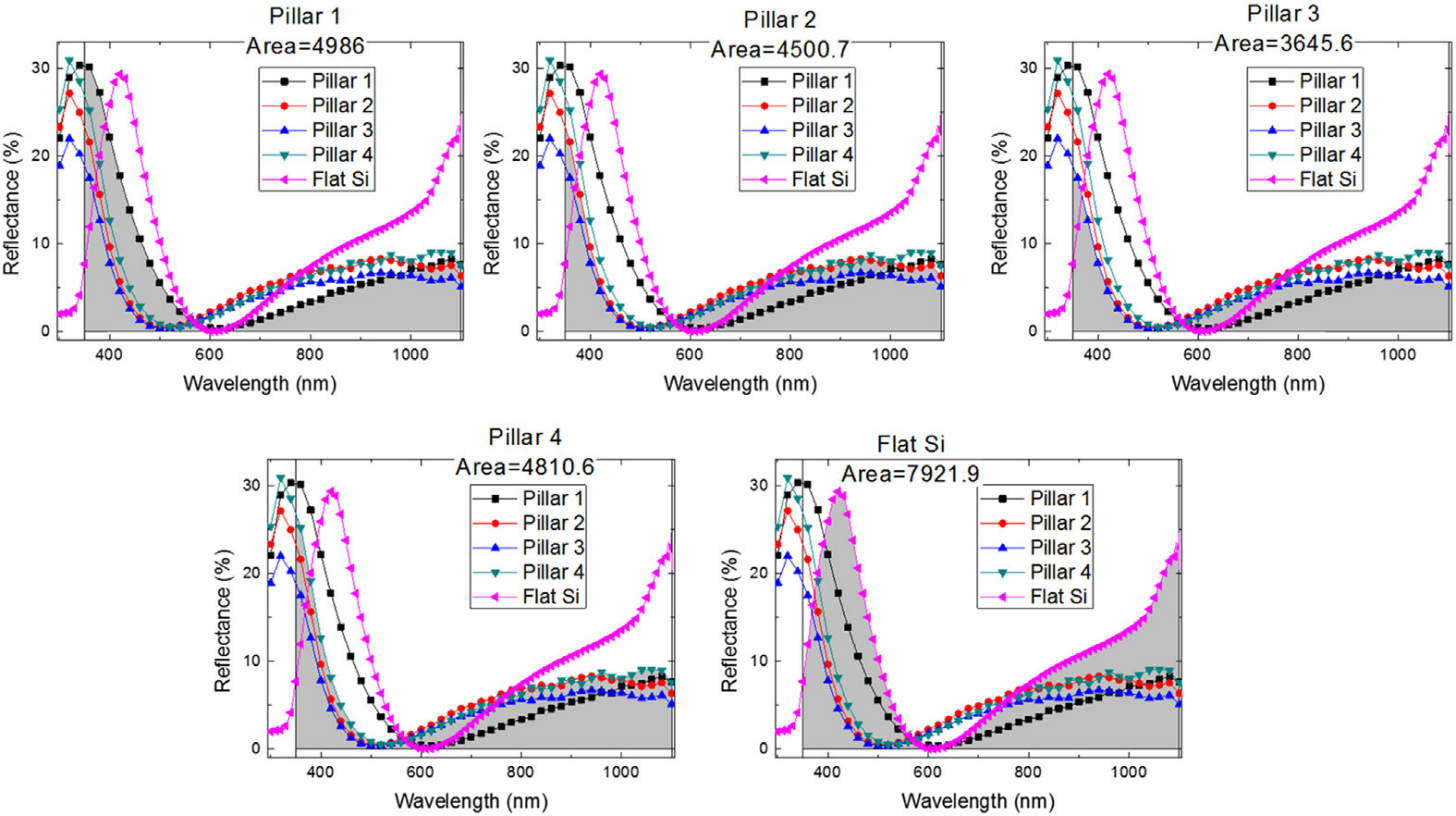
Reflectance profiles microstructure Si solar cells. Area was integrated for the photon wavelength range from 350 nm to 1100 nm. Solid gray region is shown for integration function.

**Fig. 2:**
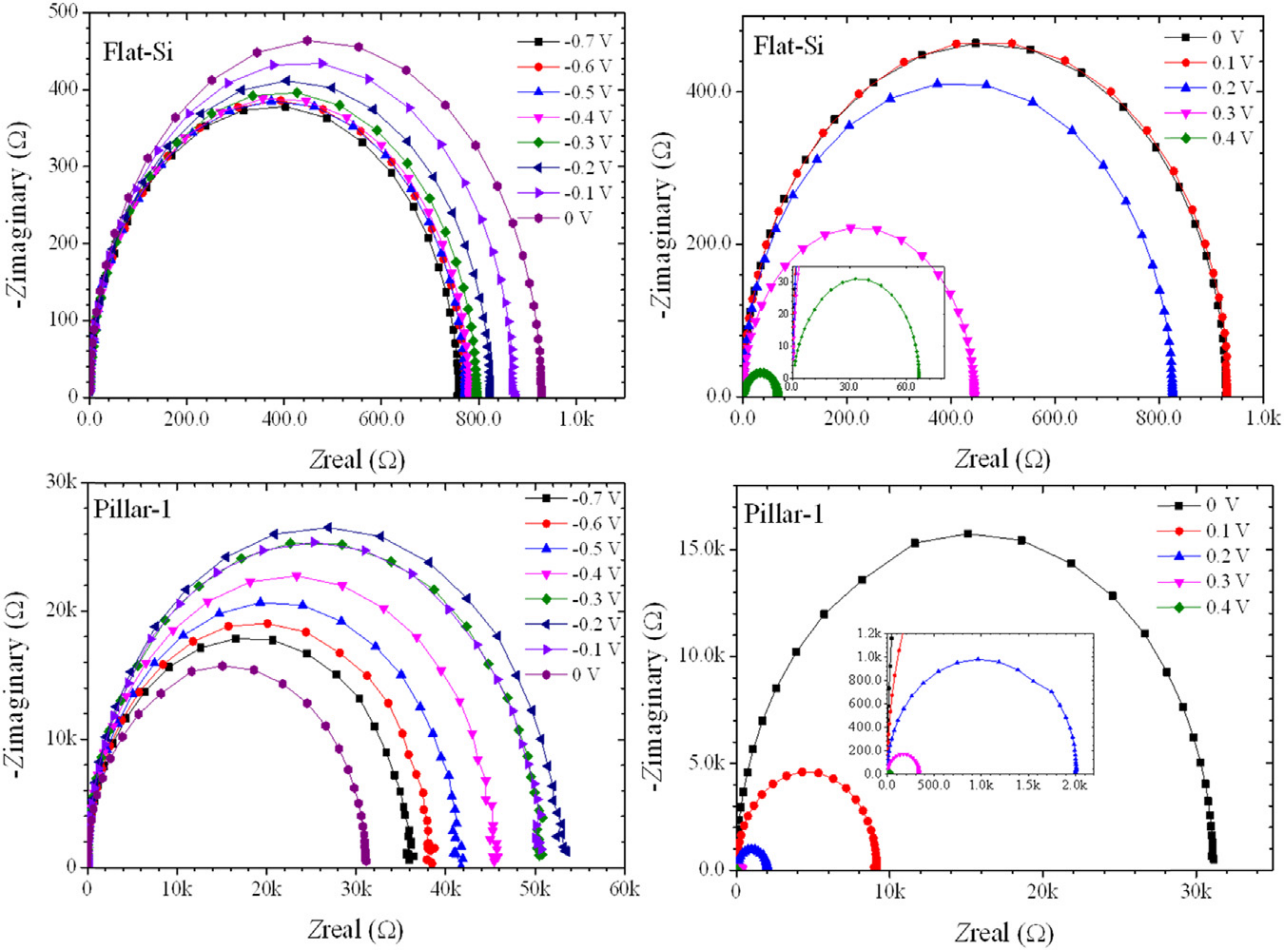
Impedance spectra of the planar sample and pillar-1 at different applied bias.

**Fig. 3:**
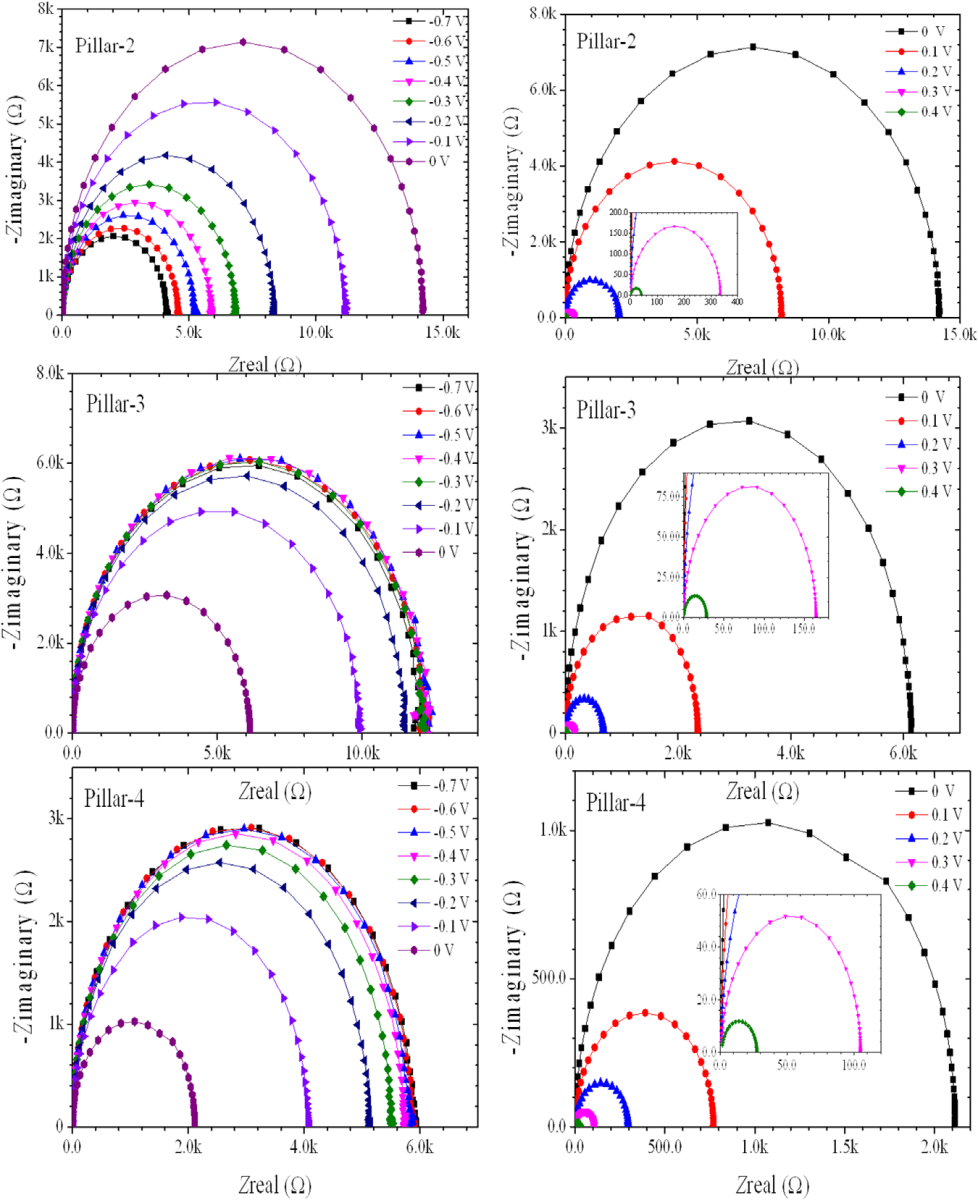
Electrochemical Impedance Spectra of pillar-2, pillar-3 and pillar-4 at different applied bias.

**Fig. 4:**
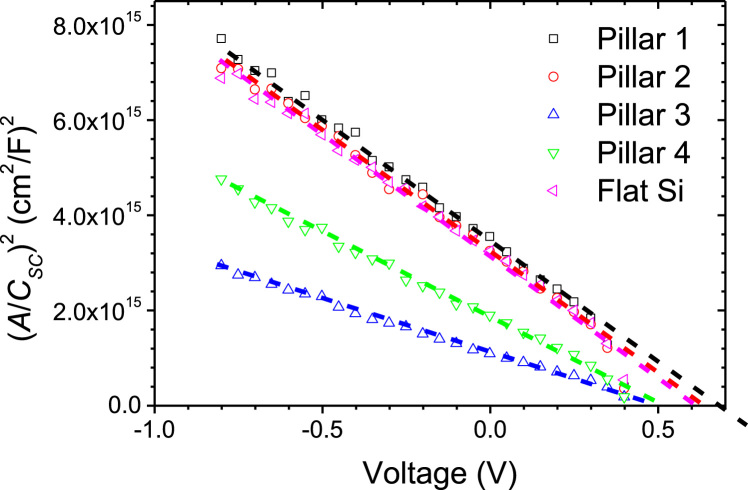
Mott Schottky plots for all the fabricated devices.

Fig. 1 shows surface reflectance of various microstructured Si solar cell, recorded by diffused integrated sphere UV-visible spectrophotometer. Microstructure Si samples are well detailed in our report [1]. Table 1 shows the integrated area under the curve of reflectance profiles. Impedance spectra of the reference flat Si device and champion pillar-1 device are shown in Fig. 2. Impedance spectra of the pillar Si devices are shown in Fig. 3. Impedance spectra of all the devices are shown for forward and reverse bias dark conditions. The Mott–Schottky characteristics of all the samples are shown in Fig. 4.

**Table 1:** Parameters of 5 μm scale Si pillar structures. Summary of integrated area under the curve of reflectance spectra, as shown in Fig. 1.

**Sample**	**Area under the curve=**$\int_{350\mathrm{nm}}^{1100\mathrm{nm}}R\left(\lambda \right)d\lambda$
Flat Si	7921.9
Pillar 1 (width=2 μm, period=4 μm, depth=5 μm)	4986.0
Pillar 2 (width=2 μm, period=7 μm, depth=5 μm)	4500.7
Pillar 3 (width=5 μm, period=7 μm, depth=5 μm)	3645.6
Pillar 4 (width=5 μm, period=10 μm, depth=5 μm)	4810.6

## 2 Experimental design, materials and methods

### 2.1 Sample preparation

The 500 µm thick p-type (100) Si wafer (Czochralski) was used as a base substrate. To form the microscale pillar structures, the photolithography mask patterns were previously formed on the Si substrate, which serve as the etching mask during the Si etching process. The exposed Si region is going to etch away. For the reactive etching, SF_6_-plasma was employed for several loops for 10 min to etch the residual polymer layer and the exposed Si parts without the PR masks.

For the formation of a p–n junction, n-type doping was done using phosphorous oxy-chloride (POCl3) source. After the formation of the n-type layer, a buffered hydrofluoric acid (5% HF) solution was used to remove the phosphosilicate glass (PSG). A thin 80 nm SiNx layer was formed over n-type layer, which actively acts as an antireflection coating and passivating layer. The size of the samples was 3.2×3.2 cm^2^ which is among one of the most efficient micro-structured solar cell with this area. The metal contacts were formed by screen printing the silver (Ag) and aluminium (Al) paste at front and back contacts, respectively, before co-firing. Planar cells with the same area but without any micro-structures were used to produce a planar junction device for the performance comparison [1].

### 2.2 Sample characterizations

Reflectance data for the fabricated samples between the wavelength ranges from 300 nm to 1100 nm are presented in Fig. 1. An integrated sphere attachment supplied with UV–vis spectrophotometer (Shimadzu-2600) was used for carrying out the diffused reflectance measurements. A necessary baseline correction was done prior to recording the reflectance spectra by using BaSO_4_ pallets. The area under the curve of reflectance profiles shown by Gray solid region is summarized in Table 1. The area under the curve was estimated over the photon wavelength range 350 nm (lower limit) to 1100 nm (upper limit). Integration function was applied for summing the finite region of interval 1 nm.

Impedance spectra of the planar and the Pillar 1 device are presented in Fig. 2. These data were measured in the dark condition for the forward bias and reverse bias conditions. The cole-cole plots for reverse bias (left) and forward bias (right) of the flat and pillar-1 devices are shown discretely. These data were recorded for the applied bias range from −0.7 V to 0.4 V with an interval of 0.1 V. These measurements were performed over the frequency range from 1 MHz to 1 Hz. Fig. 3 shows the cole-cole plots recorded for the Pillar-2, pillar-3 and pillar-4 devices. These plot shows the relation of real impedance (Z’) vs imaginary impedance (Z”).

Mott–Schottky characteristics (1/C^2^-V characteristics) obtained by using the Potentiostat/Galvanostat (ZIVE SP1, WonA Tech, Korea) is shown in Fig. 4. The Potentiostat/Galvanostat was calibrated with a standard static and dynamic circuit before the impedance and Mott–Schottky measurement. The MS measurements were performed at 20 kHz of frequency.
