# Supplementary material for: Optical properties and impedance spectroscopy analyses for microscale Si pillar solar cells
Source: Data Brief. 2017 Jul 14;14:1–5. doi: 10.1016/j.dib.2017.07.016 (PMC5522911; doi:10.1016/j.dib.2017.07.016)
Supplement: Supplementary file 1 — Supplementary material [file mmc1.docx]

***Conflicts of Interest Statement***

Re: DIB-D-17-00415

Title: Optical properties and impedance spectroscopy analyses for microscale Si pillar solar cells

We declare that this manuscript is original, has not been reported before, and is not currently being considered elsewhere. We also confirm that there is no known conflict of interest regarding this manuscript and its publication. The manuscript has been approved by all named authors.

Sincerely yours,


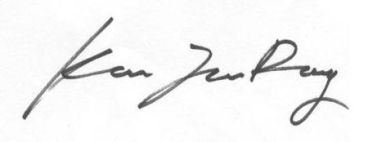


Joondong Kim

Joondong Kim, Ph.D./Professor

Department of Electrical Engineering,

Incheon National University

E-mail: joonkim@ incheon.ac.kr

Phone: +82-32-835-8770; fax: +82-32-835-0773
